# The Ion Channel Gene KCNAB2 Is Associated with Poor Prognosis and Loss of Immune Infiltration in Lung Adenocarcinoma

**DOI:** 10.3390/cells11213438

**Published:** 2022-10-31

**Authors:** Yin Lyu, Qiao Wang, Jingtian Liang, Li Zhang, Hao Zhang

**Affiliations:** 1 Thoracic Surgery Laboratory, Xuzhou Medical University, 84 West Huaihai Road, Xuzhou 221006, China; 2 Department of Thoracic Surgery, Affiliated Hospital of Xuzhou Medical University, 99 West Huaihai Road, Xuzhou 221006, China

**Keywords:** lung adenocarcinoma, KCNAB2, chemokine, immune infiltration, immunotherapy

## Abstract

The malignancy with the greatest global mortality rate is lung cancer. Lung adenocarcinoma (LUAD) is the most common subtype. The evidence demonstrated that voltage-gated potassium channel subunit beta-2 (KCNAB2) significantly participated in the initiation of colorectal cancer and its progression. However, the biological function of KCNAB2 in LUAD and its effect on the tumor immune microenvironment are still unknown. In this study, we found that the expression of KCNAB2 in tissues of patients with LUAD was markedly downregulated, and its downregulation was linked to accelerated cancer growth and poor clinical outcomes. In addition, low KCNAB2 expression was correlated with a deficiency in immune infiltration. The mechanism behind this issue might be that KCNAB2 influenced the immunological process such that the directed migration of immune cells was affected. Furthermore, overexpression of KCNAB2 in cell lines promoted the expression of CCL2, CCL3, CCL4, CCL18, CXCL9, CXCL10, and CXCL12, which are necessary for the recruitment of immune cells. In conclusion, KCNAB2 may play a key function in immune infiltration and can be exploited as a predictive biomarker for evaluating prognosis and a possible immunotherapeutic target.

## 1. Introduction

Lung cancer is the primary reason for cancer-related death in both men and women [1,2]. The most prevalent kind of lung cancer is lung adenocarcinoma, which mostly affects non-smokers [3]. Despite advancements in targeted medications and surgery [4], people with lung cancer still do not have the best survival rates with these current treatments [5]. Immunotherapy, which primarily consists of immune checkpoint inhibitors, therapeutic cancer vaccines, and adoptive cell therapies, has recently emerged as a novel treatment option for lung cancer [6]. Although the immune system’s anti-tumor response may be enhanced by these therapies, their clinical efficacy is constrained. The main reason for this is that complicated tumors influence the immune system, creating an immune-deficient tumor microenvironment that lacks immune cells with anticancer capabilities [7,8]. Therefore, to create a consistent and potent anti-tumor immune response to immunotherapy, a tumor immunological microenvironment rich in immune cells with anticancer characteristics is necessary.

Potassium channels are cellular excitatory transmembrane proteins that play an important role in tumor biology [9,10,11]. Dysregulation of potassium channel genes is associated with cancer development and patient prognosis [12], and potassium channels have been proposed as potential targets for cancer therapy [13,14]. Previous studies have shown that silencing of potassium channel genes KCNN4 and KCND2 significantly inhibits cell proliferation, migration, invasion, and tumorigenicity of lung adenocarcinoma cells [15,16,17]. However, the effect of potassium channel genes on the tumor microenvironment, especially immune cell infiltration, is still poorly understood.

The KCNAB2 gene is localized to the 1p36 region and encodes an auxiliary protein KVβ2 that modifies the properties of functional potassium voltage-gated alpha subunits [18,19]. It also encodes an aldo-keto reductase and negatively regulates members of the voltage-gated potassium channel family [20]. Some studies have found that KCNAB2 may be involved in the occurrence and development of lymphoma [21], colorectal cancer [22], pituitary tumor [23], and neuroblastoma [24], but its role in lung adenocarcinoma remains unknown.

In this study, the data from The Cancer Genome Atlas (TCGA) and Gene Expression Omnibus (GEO), and Kaplan–Meier (KM) survival curves were used to thoroughly investigate the link between KCNAB2 expression and the prognosis of LUAD patients. The enrichment analyses were performed to better understand the function of the KCNAB2 gene in LUAD. Additionally, we used TIMER and ssGSEA methods to research the relationship between KCNAB2 and immune cell infiltration. Finally, KCNAB2 was overexpressed in lung adenocarcinoma cell lines (A549 and H23) to investigate its potential biological function. Our research highlights KCNAB2’s prominent function in the development of cancer and suggests that it may be crucial for regulating immune cell infiltration in LUAD.

## 2. Materials and Methods

### 2.1. Datasets and Samples

The TCGA database (https://www.cancer.gov/, accessed on 28 March 2022) was used to retrieve the raw counts of the RNA-sequencing data, which included 535 LUAD samples and 59 nearby nontumor samples. Additionally, a total of five datasets, including GSE32863, GSE30219, GSE10072, GSE3141, and GSE13213, were collected from the GEO database (https://www.ncbi.nlm.nih.gov/gds/?term=, accessed on 9 April 2022). The primary characteristics of these five datasets are displayed in Table 1. These datasets were used to further validate the results. R software version 3.6.3 was used for the analysis. The packages “ggplot2”, “survminer”, and “survival” were used to analyze expression data and construct KM survival curves. The Log-rank tests were employed to assess the significance of prognostic differences between different groups of samples. The “ggstatsplot” was used to analyze the correlation between two groups of data. Using either Pearson’s or Spearman’s correlation, the relationship between two quantitative variables was evaluated.

### 2.2. GEPIA Database Analysis

The Gene Expression Profiling Interactive Analysis (GEPIA) (http://gepia.cancer-pku.cn/, accessed on 28 March 2022) was used to analyze the gene expression level in both cancer and healthy tissues. The expression of KCNAB2 in LUAD was assessed using GEPIA.

### 2.3. TIMER Database Analysis

The Tumor Immune Estimation Resource (TIMER) website (https://cistrome.shinyapps.io/timer/, accessed on 28 March 2022) provides systematic analysis of immune infiltration of the 32 types of tumors. With the “DiffExp” module, we evaluated the expression level of KCNAB2 in multiple types of cancer. The connection between KCNAB2 expression and the infiltration levels of six immune cells was examined using the “Gene” module. Using the “Correlation” module, the association between KCNAB2 expression and several gene marker sets of immune cells was examined.

### 2.4. Single-Sample Gene Set Enrichment Analysis

The relative enrichment fraction of 24 immune cells in LUAD was assessed by single-sample gene set enrichment analysis (ssGSEA), which was finished using “GSVA” software version 1.34.0 [25]. The Spearman’s correlation analysis was used to examine the relationship between the expression of KCNAB2 and these immune cells.

### 2.5. Kaplan–Meier Plotter Analysis

The Kaplan–Meier (KM) plotter (http://kmplot.com/analysis/, accessed on 28 March 2022) [26] is an online database that analyzes the prognosis of 21 different cancer types based on the influence of several genes. We utilized it to investigate the predictive potential of KCNAB2 expression in LUAD based on immune cells.

### 2.6. UALCAN Database Analysis

UALCAN (http://ualcan.path.uab.edu/, accessed on 28 March 2022) is a friendly web resource to analyze cancer omics data [27]. It was used in this work to detect the mRNA and protein level of KCNAB2 in LUAD [28].

### 2.7. Differentially Expressed Gene Analysis

According to the median score for KCNAB2 expression, TCGA patients with LUAD were split into two groups with high and low KCNAB2 expression. After that, using the “DESeq2” R program [29], differentially expressed gene (DEG) analysis was carried out between the two groups. The thresholds of DEGs were set at an adjusted *p*-value < 0.05 and |log2-fold-change (FC)| ≥ 1.

### 2.8. Gene Set Enrichment Analysis

Using “ClusterProfiler” [30], shown by “ggplot2”, Gene Ontology (GO) enrichment and Kyoto Encyclopedia of Genes and Genomes (KEGG) pathway studies of DEGs were carried out. When Gene Set Enrichment Analysis (GSEA) [31] was run concurrently, pathway terms with an adjusted *p*-value < 0.05 and a false discovery rate (FDR) < 0.25 were deemed statistically significantly enriched.

### 2.9. CancerSEA Database Analysis

CancerSEA is a versatile website designed to comprehensively explore the different functional states of cancer cells at the single-cell level. The database collects 72 single-cell datasets, totaling 41,900 cancer single cells from 25 human cancers, providing a map of 14 cancer-related functional states at the single-cell functional state of different cancers. In this study, it was used to investigate the average correlation between KCNAB2 and the functional status of lung adenocarcinoma cells.

### 2.10. Cell Culture

In this study, FuHeng Biology (Shanghai, China) provided the lung adenocarcinoma cell lines A549 and H23. In an incubator set to 37 °C with a humidified environment containing 5% CO_2_, cells were grown in Dulbecco’s modified Eagle’s medium (DMEM) or RPMI 1640 media (KaiJi Biology, Jiangsu, China), adding 10% fetal bovine serum and 1% penicillin/streptomycin solution.

### 2.11. Cell Transfection

GenePharma (Shanghai, China) provided the overexpression plasmid, which included the whole complementary DNA (cDNA) sequence of KCNAB2. The KCNAB2-expressing plasmid was transfected into A549 and H23 cells using Transfect-Mate transfection reagent (GenePharma, Shanghai, China).

### 2.12. Cell Proliferation and Invasion Asseys

Cells (4 × 10^3^) were planted in 96-well plates for the proliferation experiments and, at 24, 48, and 72 h, the appropriate volume of CCK-8 solution was added. The absorbance was measured at a wavelength of 450 nm. For the invasion experiment, Matrigel-coated Transwell filter inserts were used and 3 × 10^5^ cells were cultured there for 24 h in serum-free DMEM. Crystal violet (0.04%) was used to stain and count cancer cells crossing the membrane.

### 2.13. Real-Time PCR

TRIzol (Invitrogen) was used to extract the RNA and the instructions of the HiScript First Strand cDNA synthesis kit (Vazyme Biotech Co., Ltd., Nanjing, China) were followed to create the cDNA. Real-time (RT) PCR analysis was conducted according to the method described in the UltraSYBR one-step RT-qPCR kit (CWBIO, Beijing, China). In Table S1, the primers used for RT-PCR analysis were mentioned.

### 2.14. Western Blot

The whole cell lysate was prepared using the Cell Total Protein Extraction Kit (Sangon Biotech, Shanghai, China). Sodium dodecyl sulfate polyacrylamide gel electrophoresis (SDS-PAGE) was used to separate total protein and the separated protein was then transferred to 0.45 µm polyvinylidene fluoride (PVDF) membranes (MilliporeSigma, Burlington, MA, USA). After blocking with 5% non-fat milk for an hour, primary antibodies against KCNAB2 (17890-1-AP, Proteintech, Wuhan, China) and Tubulin (11224-1-AP, Proteintech, Wuhan, China) were incubated with the membranes individually for an overnight period at 4 °C. Secondary antibodies (ZB-5301, ZSGB-BIO, Beijing, China) were then used for 1 h at room temperature. The immunoreacted protein bands could then be seen. The results from Western blot were analyzed by ImageJ software version 1.8.0.

### 2.15. Immunohistochemical Staining

Immunohistochemical (IHC) staining was performed after deparaffinization in xylene and rehydration in graded ethanol. The antigen was retrieved with sodium citrate buffer (pH 6.0) at 95 °C for 20 min. A 3% H_2_O_2_ solution was used to quench endogenous peroxidase activity and a 1% bovine serum albumin buffer was used to block non-specific binding. After that, sections were incubated with the primary antibodies over night at 4 °C and then exposed to HRP-conjugated secondary antibodies for 40 min at room temperature after going through several washes. 3,3-diaminobenzidine tetrahydrochloride (DAB) was then applied to the sections. ImageJ software was used to analyze the intensity of staining.

### 2.16. Statistical Analyses

R software was used to conduct all statistical analyses. GraphPad Prism 8.0 was utilized for the statistical analysis of the data pertaining to the KCNAB2 function experiments. Student’s *t*-test was used to assess the significance of the results between the two experimental groups and one-way ANOVA was used to examine multiple group comparisons. *p* < 0.05 (*), *p* < 0.01 (**), and *p* < 0.001 (***) were significant.

## 3. Results

### 3.1. KCNAB2 Was Downregulated in LUAD

**Figure 1:**
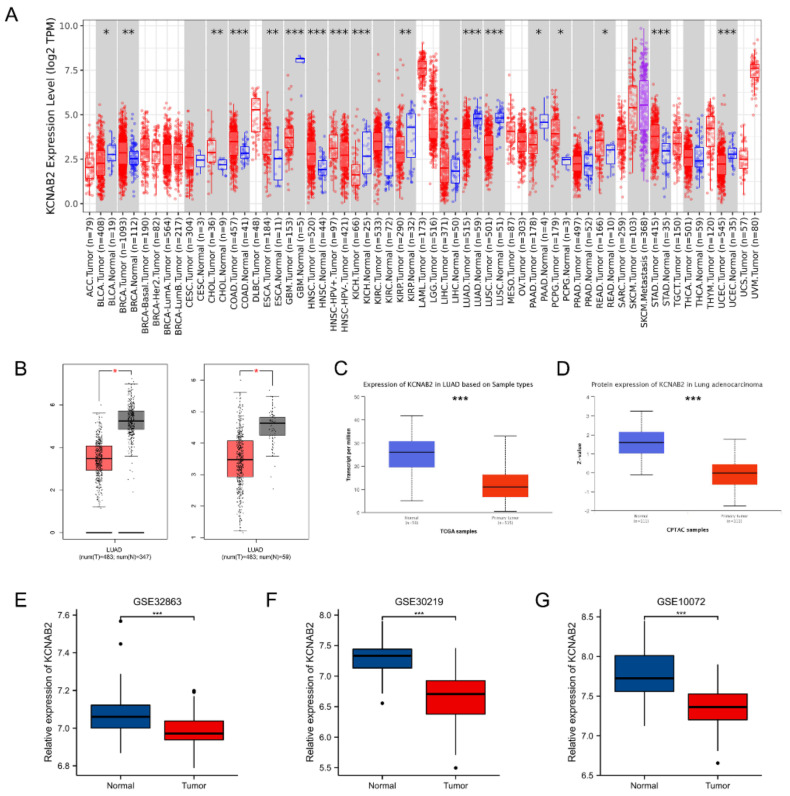
Expression of KCNAB2 in LUAD. (**A**) KCNAB2 expression levels in different tumor tissues were detected using TIMER. (**B**) Decreased expression of KCNAB2 was investigated in the GEPIA database. (**C**) The mRNA and (**D**) protein expression of KCNAB2 in LUAD were examined in the UALCAN database. (**E**–**G**) KCNAB2 mRNA expression levels were validated by GEO datasets. * *p* < 0.05, ** *p* < 0.01, *** *p* < 0.001.

First, the mRNA expression level of KCNAB2 in various cancers was detected using the TIMER online database. The findings demonstrated that KCNAB2 expression was considerably increased in nine tumor tissues and decreased in eight tumor tissues, as compared with normal tissues (Figure 1A). In the GEPIA and UALCAN databases, it was consistently found that KCNAB2’s mRNA expression was lower in LUAD tissues than in normal lung tissues (Figure 1B,C). Then, using datasets from GEO, we verified that by contrasting KCNAB2 expression in LUAD tissues with that in normal lung tissues (Figure 1E–G). To provide a comprehensive characterization of KCNAB2 expression, we detected the protein expression level of KCNAB2 using UALCAN (Figure 1D). The findings demonstrated that, compared with normal tissues, lung adenocarcinoma had considerably decreased protein expression of KCNAB2.

**Figure 2:**
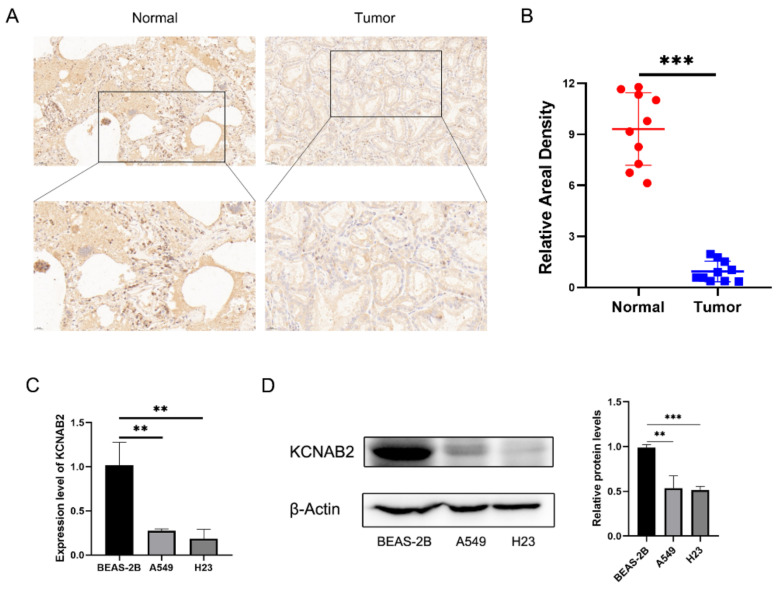
Validation of the decreased expression of KCNAB2 in LUAD using clinical tissues and cell lines. (**A**) Immunohistochemical (IHC) staining of KCNAB2 was performed in tumor tissues (n = 10) and normal tissues (n = 10). Representative images are shown. Score bars, 50 μm. (**B**) The staining was quantified. The dot plot depicts the means and standard deviation of 10 images of LUAD patient tissues and normal lung tissues. (**C**) Transcriptional levels of KCNAB2 in three different cell lines were examined by real-time PCR. (**D**) Western blot detected the protein expression level of KCNAB2 in three cell lines. ** *p* < 0.01, *** *p* < 0.001.

Further research on KCNAB2 protein expression in LUAD tissues using IHC staining revealed that, in contrast to normal lung tissues, the KCNAB2 protein level was clearly lower in tumor tissues (Figure 2A,B). Additionally, we discovered that two LUAD cell lines (A549 and H23) had much lower levels of KCNAB2 mRNA (Figure 2C) and protein (Figure 2D) expression than a lung epithelial cell line (BEAS-2B).

### 3.2. The Relationship between KCNAB2 Expression and Clinical Parameters in LUAD

**Figure 3:**
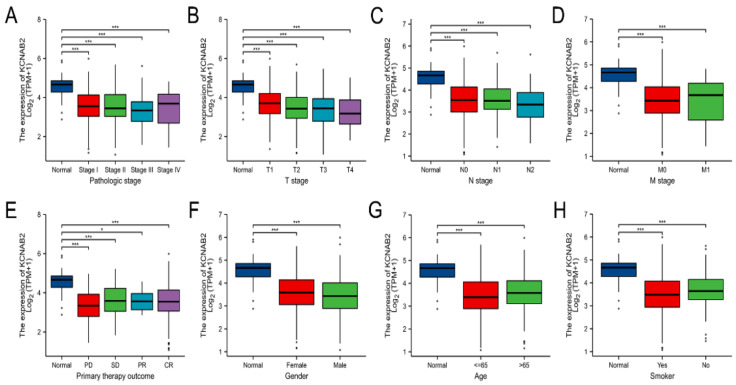
Clinical significance of KCNAB2 in LUAD. Correlation between KCNAB2 expression and clinical parameters, including (**A**) pathological stage, (**B**–**D**) TNM stage, (**E**) primary therapy outcomes, (**F**) gender, (**G**) age, and (**H**) smoker. Primary therapy outcome: PD, progressive disease; SD, stable disease; PR, partial response; CR, complete response. * *p* < 0.05, *** *p* < 0.001.

Using the TCGA data, the expression of KCNAB2 was then examined in patient groups based on several clinical indicators. According to pathological stage (Figure 3A), the expression level of KCNAB2 was significantly downregulated in LUAD samples from stage I–IV compared with the corresponding normal controls. Based on cancer TNM stage (Figure 3B–D), patients from various groups had significantly lower KCNAB2 expression levels in tumor tissues compared with normal tissues. In terms of primary therapy outcomes (Figure 3E), KCNAB2 expression was lower in patients with LUAD classified as progressive disease (PD), stable disease (SD), partial response (PR), and complete response (CR). Downregulation of KCNAB2 expression was observed in both female and male LUAD patients compared with normal controls (Figure 3F). In the tumor tissues of patients with LUAD from various age groups (Figure 3G), there was a markedly reduced KCNAB2 level according to age. Regarding smoking status (Figure 3H), both smokers and non-smokers with LUAD showed a substantial reduction in KCNAB2 expression. Furthermore, KCNAB2 expression was substantially connected with unfavorable clinicopathological outcomes, including T stage, pathologic stage, age, and gender, according to the findings of the logistic regression analysis (Table 2). These results suggest that reduced KCNAB2 expression may be positively linked with LUAD’s malignant characteristics.

### 3.3. Decreased KCNAB2 Expression Correlated with Poor Prognosis in LUAD Patients

**Figure 4:**
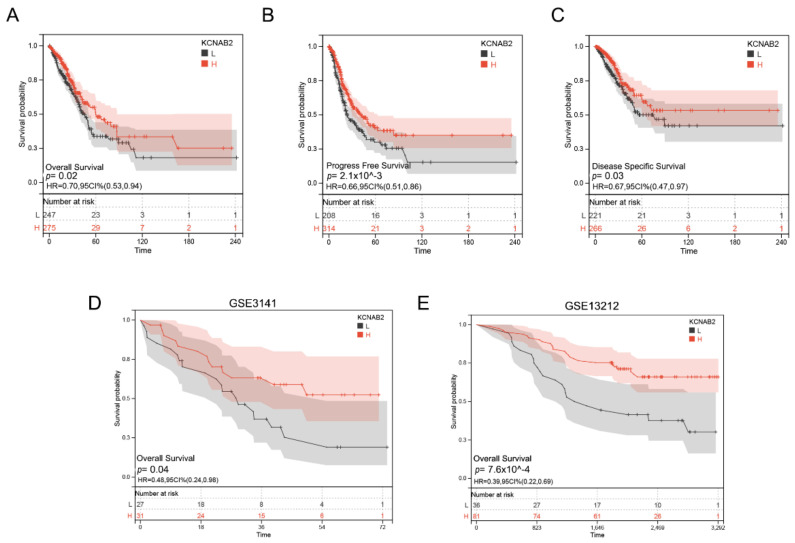
Prognostic value of KCNAB2 in LUAD. (**A**–**C**) Kaplan–Meier survival curves showed that patients with low KCNAB2 expression exhibited poor overall survival, progress free survival, and disease specific survival. (**D**,**E**) The validation of the prognostic value of KCNAB2 in LUAD by GEO datasets.

Based on the TCGA data, we analyzed the association between KCNAB2 mRNA levels and overall survival (OS) events, progress-free survival (PFS) events, and disease-specific survival (DSS) events of patients with LUAD through the KM survival curves. According to the results, poor OS, PFS, and DSS were observed to be correlated with reduced KCNAB2 expression (Figure 4A–C). Our analysis of GEO datasets further validated the prognostic value of KCNAB2 in LUAD and discovered that its downregulation was associated with poor outcomes (Figure 4D,E).

### 3.4. Survival Analysis of KCNAB2 in Different Clinical Subgroups

**Figure 5:**
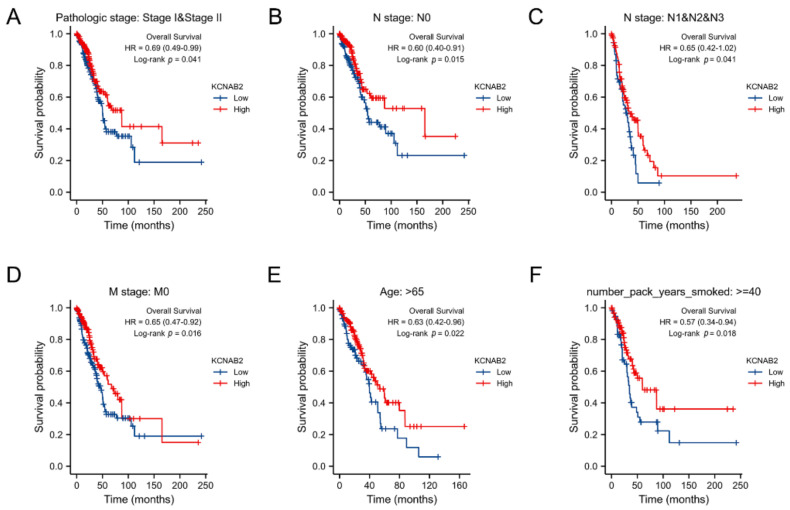
Associations between the expression of KCNAB2 and the overall survival based on LUAD patients with different clinical parameters. (**A**) Stage I–II, (**B**) N0, (**C**) N1–N3, (**D**) M0, (**E**) age over 65 years old, and (**F**) smoker over 40 years.

The association between KCNAB2 expression and OS was later established across several subgroups by clinical characteristics in order to confirm the prognosis of KCNAB2 in TCGA patients with LUAD. The research revealed that pathological stage I–II (HR = 0.69, *p* = 0.041), N0 (HR = 0.60, *p* = 0.015), N1–N3 (HR = 0.65, *p* = 0.041), M0 (HR = 0.65, *p* = 0.016), patients over 65 years old (HR = 0.63, *p* = 0.022), and smoker over 40 years old (HR = 0.57, *p* = 0.018) were all associated with low KCNAB2 expression and a bad outcome in LUAD (Figure 5A–F).

### 3.5. Identification of DEGs and Functional Enrichment Analysis

**Figure 6:**
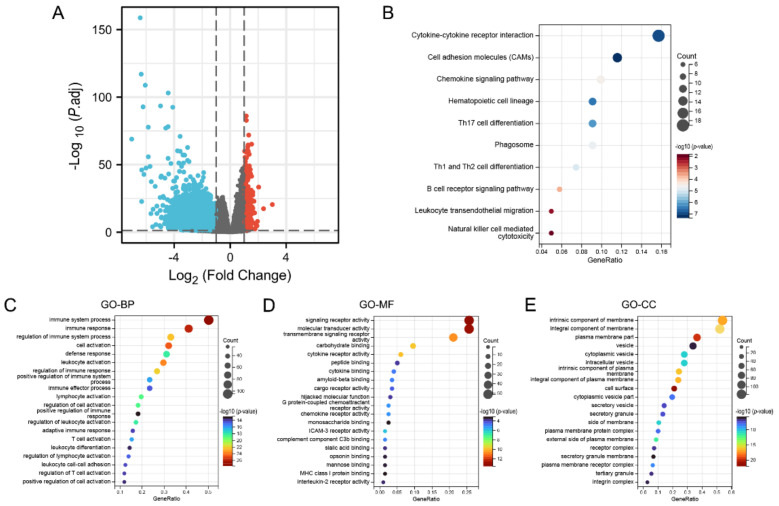
KCNAB2−related differentially expressed genes (DEGs) and functional enrichment analysis using GO and KEGG. (**A**) Volcano plot of DEGs. Blue and red dots indicate the significantly downregulated and upregulated DEGs. (**B**) Top 10 terms of KEGG analysis of upregulated DEGs. (**C**–**E**) Top 20 terms of GO analysis of DEGs, including biological process (BP), molecular function (MF), and cellular component (CC).

In summary, KCNAB2 may affect the prognosis of patients with LUAD. To explore the specific mechanisms by which KCNAB2 affects prognosis, KCNAB2-associated differentially expressed genes were explored first. The two groups of TCGA patients with LUAD with high and low KCNAB2 expression were separated based on the median score for KCNAB2 expression. Through the “DESeq2” method, there were 792 genes that showed differential expression (adjusted *p*-value < 0.05, |log2 FC| ≥ 1), including 550 downregulated genes and 242 upregulated genes (Figure 6A and Table S2). Then, to investigate the pathways and biological activities connected to KCNAB2, KEGG and GO enrichment analyses were performed on a total of 242 elevated genes (Table S3). The top 10 terms from KEGG and the top 20 terms from each part of the GO enrichment analysis are shown (Figure 6B–E). Notably, numerous immune-related pathways, such as cytokine–cytokine receptor interaction, chemokine signaling pathway, B cell receptor signaling pathway, leukocyte transendothelial migration, Th17 cell differentiation, Th1 and Th2 cell differentiation, and natural killer cell mediated cytotoxicity in LUAD, were found to be strongly associated with KCNAB2 among these pathways. In addition, immune system process, leukocyte activation, leukocyte differentiation, T cell activation, and positive regulation of immune response in LUAD were all immune-response-related activities where KCNAB2 was enriched in terms of the biological process.

**Figure 7:**
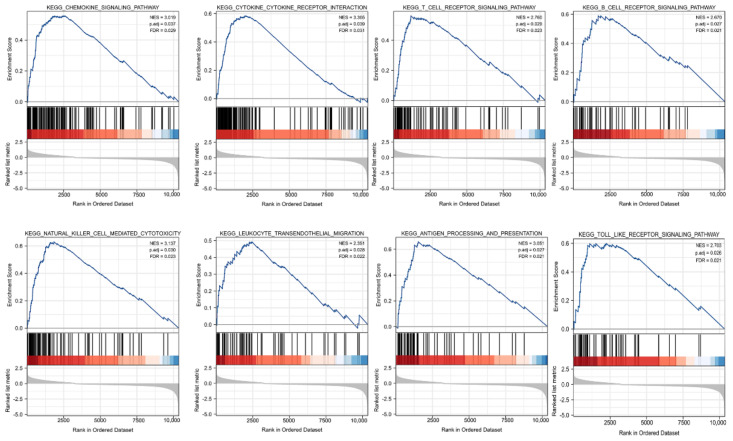
Identification of KCNAB2−related signaling pathways in LUAD using GSEA.

GSEA was carried out to better investigate the molecular processes impacted by KCNAB2 in TCGA patients with LUAD. Similar to the results of KEGG enrichment analysis, numerous immune functional gene sets were shown to be enriched in LUAD by GSEA (Figure 7 and Table S4), including those involved in cytokine–cytokine receptor interactions, natural killer cell mediated cytotoxicity, chemokine signaling pathways, antigen processing and presentation, T cell receptor signaling pathway, Toll-like receptor signaling pathway, B cell receptor signaling pathway, and leukocyte transendothelial migration. These findings overwhelmingly pointed to the involvement of KCNAB2 in the control of the immune response in LUAD.

### 3.6. Immune Cell Infiltration and the Expression of KCNAB2

**Figure 8:**
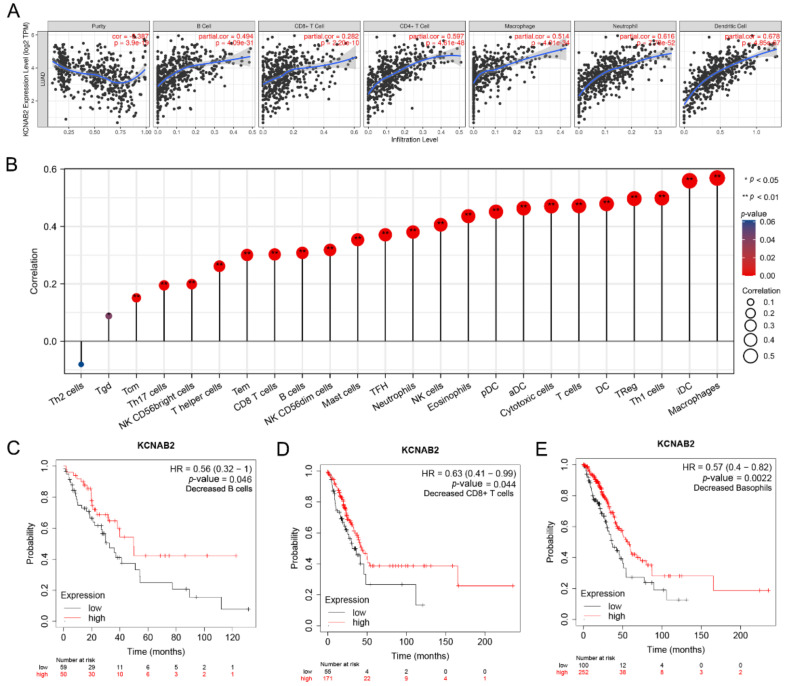
The relationship between KCNAB2 expression an immune cell infiltration. (**A**) The expression level of KCNAB2 is positively correlated with the infiltration of different immune cells using the TIMER database. (**B**) KCNAB2 expression has a significant correlation with the infiltration of immune cells in LUAD using the ssGSEA method. (**C**–**E**) Correlations between KCNAB2 expression and overall survival in different immune cell subgroups in LUAD patients were estimated by Kaplan–Meier plotter.

The effectiveness of anti-cancer treatments and patient outcomes are significantly influenced by immune infiltration in the tumor microenvironment. Using the TIMER online tools, we detected the association between KCNAB2 expression and six different subtypes of infiltrating immune cells, including B cells, CD8+ T cells, CD4+ T cells, macrophages, neutrophils, and dendritic cells. The findings demonstrated a substantial positive correlation between the infiltration of these six immune cells and the expression level of KCNAB2 in LUAD (Figure 8A).

Next, we assessed the relationship between KCNAB2 and immune infiltration using ssGSEA to further evaluate the impact of KCNAB2 on the tumor microenvironment. The results revealed that KCNAB2 expression was strongly correlated with 18 types of infiltrating immune cells out of 24 types (Spearman r > 0.3, *p*-value < 0.05) (Figure 8B). Among them, cytotoxic cells, CD8+ T cells, natural killer cells, B cells, and dendritic cells are all immune cell types that are positively associated and have anti-cancer characteristics. 

The expression level of KCNAB2 is highly connected with immune infiltration and a bad prognosis in LUAD, so we further explored whether KCNAB2 expression influences the prognosis of LUAD as a result of immune infiltration. On the basis of the KCNAB2 expression level in LUAD in relevant immune cell subgroups, we conducted prognostic analyses using the KM plotter database. The results suggested that patients with LUAD who exhibited reduced infiltration of B cells, CD8+ T cells, and basophils, as well as low KCNAB2 expression, had poor prognoses (Figure 8C–E and Figure S1). This would also imply that KCNAB2 may be more closely associated with these three immune cells than with other immune cells. These findings suggest that KCNAB2 downregulation may be followed by a decreased anti-cancer immune infiltrate, which would lead to a poorer prognosis for survival.

### 3.7. The Link between KCNAB2 Expression and Immune Cell Markers

We used the TIMER database to identify the expression connection of KCNAB2 with immune cell biomarkers in LUAD in order to further investigate the function of KCNAB2 in tumor immunity. As shown in Table 3, KCNAB2 strongly linked favorably with immune cell indicators in LUAD. When analyzing immune infiltration in cancer tissues, tumor purity is a crucial factor. After accounting for tumor purity, KCNAB2 expression was shown to be substantially correlated with the majority of immunological markers in various immune cell types in LUAD. These results provide additional evidence that the expression of KCNAB2 is highly correlated with immune infiltration and support the notion that KCNAB2 plays a critical part in immune escape in the microenvironment of LUAD.

### 3.8. KCNAB2 Upregulated the Expression of Chemokines

**Figure 9:**
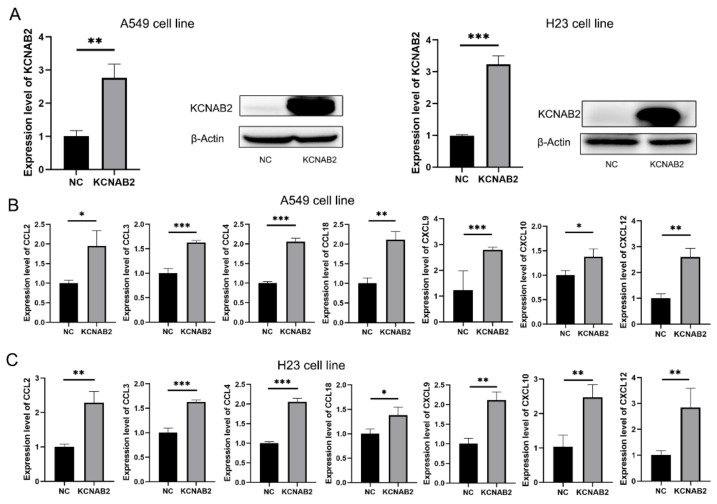
Real-time PCR results for analyzing the expression of tumor immune-related chemokines. (**A**) The overexpression effect of KCNAB2 in A549 cell line and H23 cell line was verified at the mRNA and protein levels. (**B**,**C**) Chemokines, including CCL2, CCL3, CCL4, CCL18, CXCL9, CXCL10, and CXCL12, were upregulated by KCNAB2 in A549 (**B**) and H23 cell lines (**C**). * *p* < 0.05, ** *p* < 0.01, *** *p* < 0.001.

Based on the findings of GSEA and immune infiltration analyses, we proposed that KCNAB2 may primarily influence the immunological environment by influencing immune cell movement. Chemokines are important to the migration of immune cells, so we explored how KCNAB2 interacts with chemokines that are relevant to cancer immunity. The chemokines are listed in Table S5. After transfecting KCNAB2-overexpressing plasmids into A549 and H23 cell lines (Figure 9A), we discovered that the mRNA expression of CCL2, CCL3, CCL4, CCL18, CXCL9, CXCL10, and CXCL12 was increased in these two cell lines (Figure 9B,C). In addition to this, we also examined the effect of KCNAB2 on the functional status of the cancer cells. The results of cell proliferation and invasion assay showed that overexpression of KCNAB2 could promote the cell proliferation (Figure S2A,B) and cell invasion (Figure S2C,D) ability of LUAD to a certain extent, but the difference was not statistically significant, which was consistent with the results observed in the cancerSEA database (Figure S2E).

## 4. Discussion

The KCNAB2 gene encodes KVβ2, an auxiliary protein that alters the characteristics of functioning potassium voltage-gated alpha subunits. According to several studies, KCNAB2 may play a role in the occurrence and progression of neuroblastoma, glioblastoma multiforme, pituitary tumor, and lymphoma. We chose to carry out a completely integrated bioinformatics study to pinpoint the KCNAB2 gene’s related functions and pathways in LUAD, because there had not been many studies on this gene’s role in cancer.

The KCNAB2 gene’s expression and prognostic significance were initially investigated in this work, and it was discovered that the expression level of KCNAB2 was decreased in LUAD. Poor outcomes in LUAD are linked with decreased KCNAB2 expression. At the same time, bad clinicopathological features are associated with low KCNAB2 expression. These findings confirmed the possibility that KCNAB2 functions as a standalone predictive biomarker in LUAD and might support the advancement of targeted precision oncology.

The state of tumor immune infiltration is crucial in the process of tumor immunotherapy [32]. In this study, we carried out functional enrichment analyses to examine the molecular mechanisms underlying KCNAB2-mediated LUAD progression and poor prognosis. Interestingly, we discovered that the immune-related processes, such as immune system process, leukocyte activation, leukocyte differentiation, and T cell activation, as well as pathways such as cytokine–cytokine receptor interactions, natural-killer-cell-mediated cytotoxicity, and chemokine signaling pathways, were notably enriched in the group with high KCNAB2 expression. The results of GSEA also supported this judgment. Next, we used the TIMER and ssGSEA to validate the correlations between KCNAB2 expression and other immunological markers in LUAD to deeply comprehend the crosstalk with the immune response. For the first time, our investigation revealed a strong positive connection between KCNAB2 expression and the tumor infiltration of 18 immune effector cells from the innate and adaptive immune systems. Among them, innate immune cells, such as mast cells, dendritic cells, NK cells, macrophages, and neutrophils, are important for tumor monitoring and repression [33,34]. In addition, the adaptive immune cells, including CD8+ T cells, T helper cells, T memory cells, cytotoxic T cells, and B cells, are immunological effector cells and are essential for anti-cancer immunotherapy [35,36]. At the same time, it is noteworthy that there are also immune cells that are considered to be tumor-promoting among these 18 immune cells, such as Treg, which does not seem to be consistent with the purpose of our study. However, Overacre-Delgoffe, A. E. et al. found that Tregs can be classified into three categories: stable Treg, unstable Treg, and fragile Treg. Fragile Treg is thought to have anti-tumor activity owing to its decreased expression of immunosuppressive factors such as interleukin 10 (IL-10) and its ability to secrete interferon-γ (IFN-γ) [37,38]. Whether tumor cells overexpressing KCNAB2 can predominantly recruit fragile Treg or whether the secreted cytokines can drive the fragile phenotype of Treg are questions that need to be further explored. Based on these findings, we hypothesized that KCNAB2 downregulation may be associated with diminished immune cell infiltration, and thus decreased sensitivity to immunotherapy. More interactive research is required to comprehend how KCNAB2 overexpression in lung cancer cells influences the patterns of immune cell infiltration.

Based on the findings of GSEA, we inferred that KCNAB2 may regulate the immune microenvironment mainly by affecting the migration process of immune cells. Chemokines have been shown to be crucial for the directed migration of immune cells in earlier research [39,40,41]. We thus detected the connections between KCNAB2 expression and cancer-related chemokines. In the A549 cell line, we discovered that overexpressing KCNAB2 could increase the messenger RNA expression of CCL2, CCL3, CCL4, CCL18, CXCL9, CXCL10, and CXCL12. The H23 cell line produced similar results. A recent study by Du et al. demonstrated the therapeutic potential of CCL2 in adoptive cell therapy. Their findings suggest that the CCL2/CCR2 axis enhances the migration of CAR-T cells into brain metastases of non-small cell lung cancer [42]. Xue et al. showed that CCL4 was able to recruit CD103+ dendritic cells, thereby activating CD8+ T cells [43]. CXCR3, which is the receptor for the chemokines CXCL9 and CXCL10, is expressed by effector CD8+ T cells, Th1 cells, and NK cells. In response to these chemokines, these cells can move into tumors [44]. The Th17 cells, which have stem-like qualities [45] and mediate powerful antitumor immunity [46], may be transported into tumors with the help of the CXCL12/CXCR4 axis. At the same time, we also examined the effect of KCNAB2 on the functional status of lung adenocarcinoma cells. KCNAB2 could promote cell proliferation and invasion to some extent, but the difference was not statistically significant. Similar results were observed with the cancerSEA database. It is worth noting that KCNAB2 does not seem to have a significant effect on the functional status of lung adenocarcinoma cells themselves, as predicted from the cancerSEA database, which motivates us to explore the effect of KCNAB2 on the tumor microenvironment.

However, even though we employed many databases for cross-validation and performed a comprehensive investigation on KCNAB2, our study had certain drawbacks. First, the diagnostic and prognostic usefulness of KCNAB2 in lung squamous cell carcinomas (LUSC), large cell lung carcinomas (LCLC), and small cell lung carcinomas (SCLC) was not examined in this investigation. Second, in the current study, the majority of the analyses were conducted using the mRNA levels of KCNAB2. The findings would be more persuasive if they were subjected to a more thorough study based on protein expression levels. Third, we only preliminarily verified the effect of KCNAB2 on chemokine expression in vitro. We lack direct evidence of KCNAB2 being involved in tumor immune infiltration. Future studies into these problems are worthwhile.

## 5. Conclusions

In conclusion, our research revealed that the expression of KCNAB2 is markedly decreased and is strongly associated with the dismal prognosis of LUAD patients. Additionally, we investigated possible evidence indicating that KCNAB2 affects the infiltrating level of immune cells in the tumor environment of patients with LUAD. Therefore, these discoveries have the potential to further our understanding of KCNAB2’s function as well as its prospective use in cancer immunotherapy and prognosis.

## Figures and Tables

**Table 1:** The main features of the five selected datasets used in this study.

GEO Datasets	Platform	Sample Size		Publication Years
		LUAD	Normal	
GSE32863	GPL6884	58	58	2012
GSE30219	GPL570	85	14	2013
GSE10072	GPL96	58	47	2008
GSE3141	GPL570	58	0	2005
GSE13213	GPL6480	117	0	2009

**Table 2:** The results of the logistic regression analysis of KCNAB2.

Characteristics	Total (N)	Odds Ratio (OR)	*p*-Value
T stage (T2, T3, and T4 vs. T1)	532	0.658 (0.522–0.824)	<0.001
N stage (N1, N2, and N3 vs. N0)	519	0.890 (0.714–1.109)	0.299
M stage (M1 vs. M0)	386	0.982 (0.612–1.592)	0.940
Pathologic stage (Stage III and Stage IV vs. Stage I and Stage II)	527	0.764 (0.591–0.984)	0.037
Age (>65 vs. <=65)	516	1.284 (1.043–1.588)	0.019
Gender (Male vs. Female)	535	0.799 (0.649–0.982)	0.034
Smoker (Yes vs. No)	521	0.741 (0.546–0.999)	0.052

**Table 3:** Correlation analysis between KCNAB2 and gene markers of immune cells.

Immune Cells	Gene Markers	None		Purity	
		Correlation	*p*-Value	Correlation	*p*-Value
B cell	CD19	0.439	***	0.331	***
	CD79A	0.371	***	0.257	***
T cell (general)	CD3D	0.471	***	0.346	***
	CD3E	0.587	***	0.498	***
	CD2	0.59	***	0.499	***
CD8+ T cell	CD8A	0.48	***	0.381	***
	CD8B	0.394	***	0.306	***
Monocyte	CD86	0.675	***	0.616	***
	CSF1R	0.727	***	0.685	***
TAM	CCL2	0.416	***	0.328	***
	CD68	0.674	***	0.636	***
	IL10	0.573	***	0.493	***
M1	IRF5	0.627	***	0.587	***
	PTGS2	-0.123	*	-0.143	**
	NOS2	0.232	***	0.167	***
M2	CD163	0.673	***	0.63	***
	VSIG4	0.624	***	0.58	***
	MS4A4A	0.628	***	0.572	***
Neutrophils	CEACAM8	0.286	***	0.289	***
	ITGAM	0.73	***	0.7	***
	CCR7	0.567	***	0.477	***
Natural killer cell	KIR2DL1	0.195	***	0.144	**
	KIR2DL3	0.248	***	0.178	***
	KIR2DL4	0.209	***	0.134	**
	KIR3DL1	0.238	***	0.183	***
	KIR3DL2	0.313	***	0.248	***
	KIR3DL3	0.077	ns	0.057	ns
	KIR2DS4	0.227	***	0.165	***
Dendritic cell	HLA-DPB1	0.638	***	0.582	***
	HLAD-QB1	0.485	***	0.407	***
	HLA-DRA	0.573	***	0.503	***
	HLA-DPA1	0.612	***	0.557	***
	CD1C	0.384	***	0.316	***
	NRP1	0.203	***	0.172	***
	ITGAX	0.804	***	0.777	***

NS, *p* > 0.05, * *p* < 0.05, ** *p* < 0.01, *** *p* < 0.001.

## Data Availability

All of the data corresponding to the LUAD series used in this study are available in TCGA and GEO databases, which are both public functional genomics data repositories.

## Supplementary Materials

The following supporting information can be downloaded at: https://www.mdpi.com/article/10.3390/cells11213438/s1, Figure S1: Correlations between KCNAB2 expression and overall survival in different immune cell subgroups in LUAD patients were estimated by KM plotter; Figure S2: KCNAB2 regulates LUAD cell proliferation and cell invasion; Table S1: The primers used in this study; Table S2: Differentially expressed genes related to KCNAB2; Table S3: The results of GO and KEGG enrichment analysis based on 242 upregulated genes; Table S4: The results of GSEA; Table S5: The chemokines in this study.
