# Supplementary material for: The Ion Channel Gene KCNAB2 Is Associated with Poor Prognosis and Loss of Immune Infiltration in Lung Adenocarcinoma
Source: Cells. 2022 Oct 31;11(21):3438. doi: 10.3390/cells11213438 (PMC9653610; doi:10.3390/cells11213438)
Supplement: Supplementary file 1 [file cells-11-03438-s001.zip › cells-1931622-supplementary.pdf]

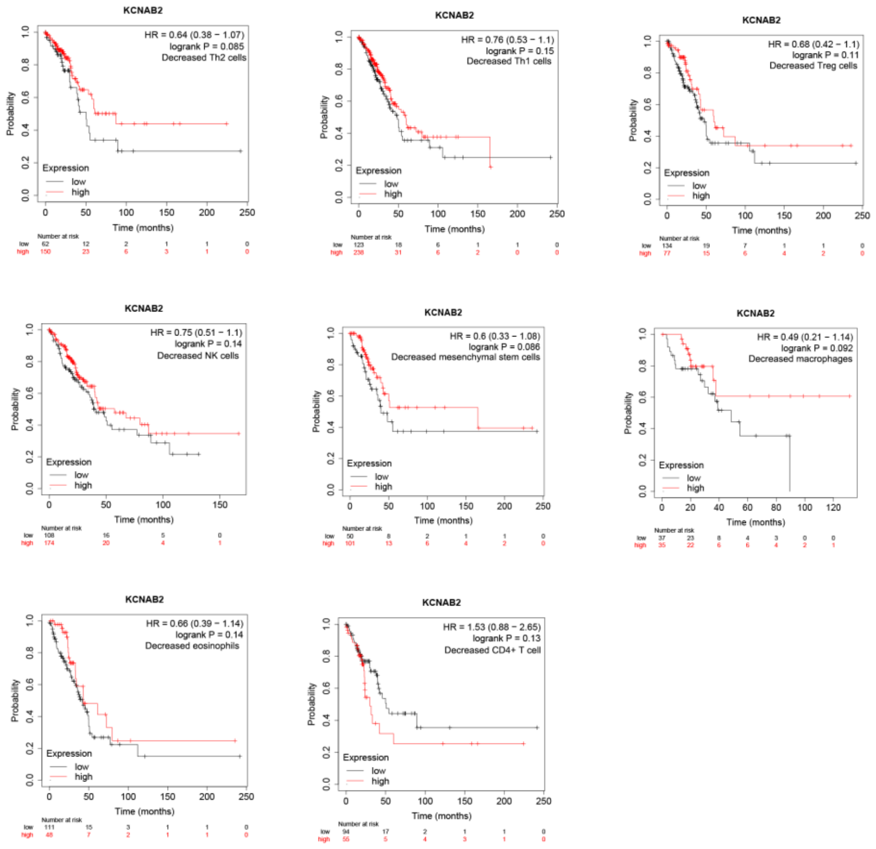

Supplementary Figure S1. Correlations between KCNAB2 expression and overall survival in different immune cell subgroups in LUAD patients were estimated by Kaplan-Meier plotter.

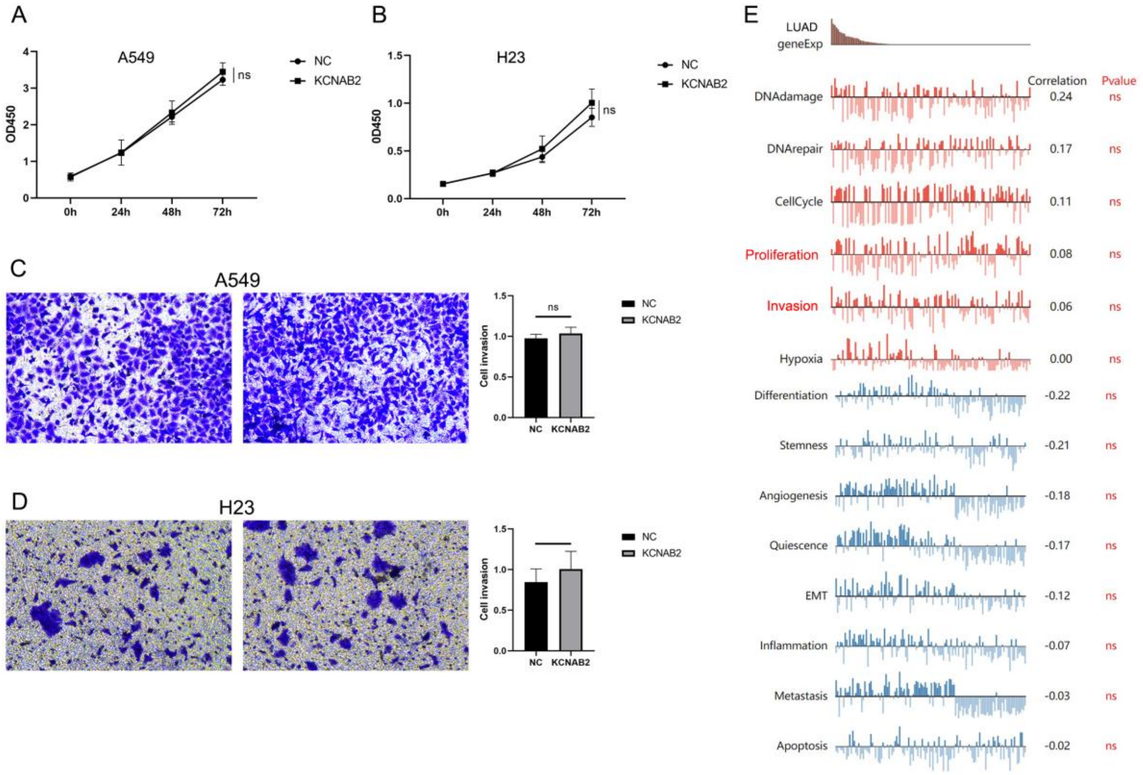

Supplementary Figure S2. KCNAB2 regulates LUAD cell proliferation and cell invasion. (**A, B**) CCK8 detects the effect of overexpression of KCNAB2 on the proliferation of A549 (A) and H23 (B) cells. (**C, D**) Transwell assay detects the effect of overexpression of KCNAB2 on the invasion of A549 (C) and H23 (D) cells. (**E**) The cancerSEA database was used to analyse the correlation between KCNAB2 and the functional state of lung adenocarcinoma cells.
